# Caveolin-1 scaffolding domain peptide regulates glucose metabolism in lung fibrosis

**DOI:** 10.1172/jci.insight.137969

**Published:** 2020-10-02

**Authors:** Venkadesaperumal Gopu, Liang Fan, Rashmi S. Shetty, M.R. Nagaraja, Sreerama Shetty

**Affiliations:** Texas Lung Injury Institute, Department of Medicine, University of Texas Health Science Center at Tyler (UTHCT), Tyler, Texas, USA.

**Keywords:** Pulmonology, Fibrosis, Glucose metabolism, P53

## Abstract

Increased metabolism distinguishes myofibroblasts or fibrotic lung fibroblasts (fLfs) from the normal lung fibroblasts (nLfs). The mechanism of metabolic activation in fLfs has not been fully elucidated. Furthermore, the antifibrogenic effects of caveolin-1 scaffolding domain peptide CSP/CSP7 involving metabolic reprogramming in fLfs are unclear. We therefore analyzed lactate and succinate levels, as well as the expression of glycolytic enzymes and hypoxia inducible factor-1α (HIF-1α). Lactate and succinate levels, as well as the basal expression of glycolytic enzymes and HIF-1α, were increased in fLfs. These changes were reversed following restoration of p53 or its transcriptional target microRNA-34a (miR-34a) expression in fLfs. Conversely, inhibition of basal p53 or miR-34a increased glucose metabolism, glycolytic enzymes, and HIF-1α in nLfs. Treatment of fLfs or mice having bleomycin- or Ad-TGF-β1–induced lung fibrosis with CSP/CSP7 reduced the expression of glycolytic enzymes and HIF-1α. Furthermore, inhibition of p53 or miR-34a abrogated CSP/CSP7-mediated restoration of glycolytic flux in fLfs in vitro and in mice with pulmonary fibrosis and lacking p53 or miR-34a expression in fibroblasts in vivo. Our data indicate that dysregulation of glucose metabolism in fLfs is causally linked to loss of basal expression of p53 and miR-34a. Treatment with CSP/CSP7 constrains aberrant glucose metabolism through restoration of p53 and miR-34a.

## Abstract 2

Caveolin-1-derived peptide CSP7 inhibits existing lung fibrosis through glycolytic inhibition and restoration of baseline p53 expression in activated fibrotic lung fibroblasts.

## Graphical abstract

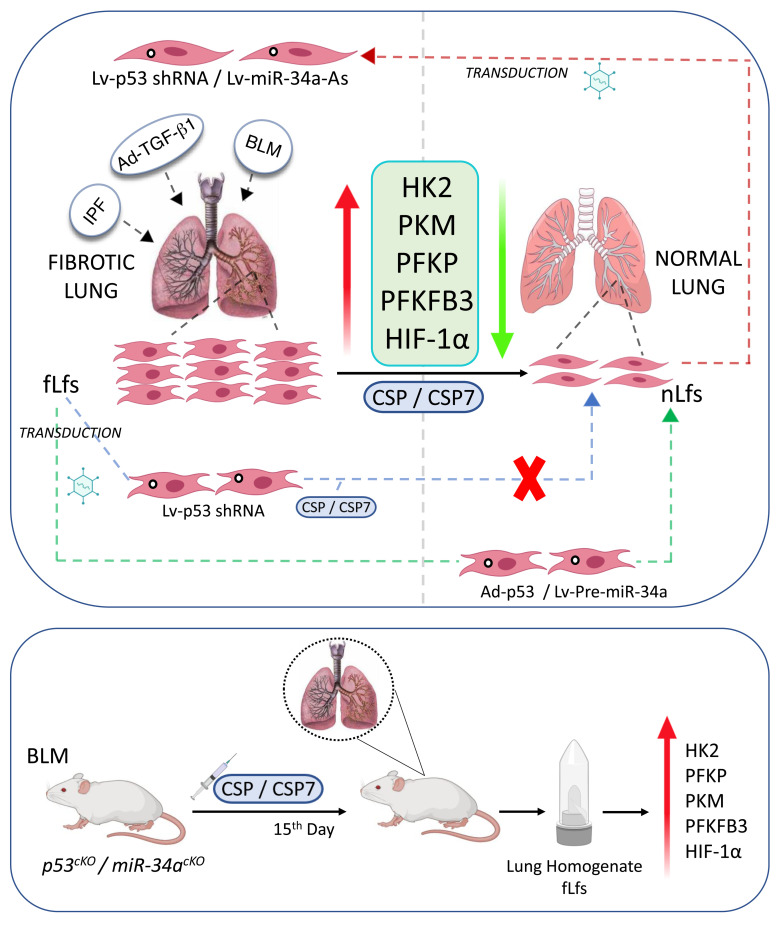

## Introduction

Glucose metabolism begins with its conversion to pyruvate through sequences of biochemical reactions termed glycolysis. During aerobic respiration, pyruvate is transported into mitochondria to be converted into CO_2_ and the acetyl portion of acetyl coenzyme A. This process produces nicotinamide adenine dinucleotide (NADH), which later goes through oxidative phosphorylation (1–3). However, under anaerobic conditions, pyruvate is converted to lactate in the cytoplasm (4, 5). Increased conversion of glucose to lactate can occur under both aerobic and anaerobic conditions. One of the distinctions of cancer cells is that they metabolize glucose by glycolysis, whereas noncancerous cells metabolize glucose by oxidative phosphorylation (1, 6). The major shift to aerobic glycolysis with lactate production, with increased uptake of glucose, is likely used by proliferating carcinoma cells to promote the efficient conversion of glucose into the macromolecules required for increased cellular mass (7, 8). This metabolic change, termed the Warburg effect, is a key event during tumor progression (9, 10). Increased metabolic activation and production of lactate has been reported in tumors surrounding stromal fibroblasts, which nourish tumor cells. Increased aerobic glycolysis in stromal cells results in increased production and secretion of lactate. The lactate is then transferred to adjacent cancer cells and converted to pyruvate, which functions as a substrate for mitochondrial oxidative phosphorylation (1, 6). A relative deficiency of caveolin-1 (CAV1) in tumor-associated fibroblasts has been postulated to underlie the reverse Warburg effect, in which cancer cell oxidants downregulate CAV1 in stromal fibroblasts that are tumorigenic in breast cancers (11, 12).

Interstitial lung diseases (ILDs) — including one of its most common types, idiopathic pulmonary fibrosis (IPF) — are progressive and debilitating lung diseases. These diseases are fatal, with a postdiagnosis median survival of less than 3 years (13, 14). Activated myofibroblasts are important contributors for normal wound repair processes that eventually undergo apoptosis. However, activated myofibroblasts or fibrotic lung fibroblasts (fLfs) in IPF lungs resist apoptosis and exhibit excessive proliferation and migration. This leads to a progressive accumulation of these cells. Since diagnosis of IPF is often in the advanced stages of disease development, there is a strong consensus to target fLfs to effectively mitigate IPF and other progressive fibrotic disorders. Multiple recent reports have indicated higher glycolytic activity in fibrotic areas and fLfs isolated from IPF lungs. These studies have implicated a persistent state of activation of fLfs to upregulated key glycolytic enzymes (15, 16). Metabolic reprogramming of fLfs has been implicated in the pathogenesis of IPF. Glucose transporter proteins, particularly GLUT1, play an important role in metabolic activation of cells. GLUT1 expression is regulated by changes in metabolic state and oxidative stress, which involve signaling molecules such as cyclic adenosine monophosphate (cAMP), p53, PI3-kinase, and AKT (17–20). Inhibition of GLUT1 reduced α-SMA expression in lung fibroblasts (21), indicating a direct link between glycolytic activation and profibrogenic transformation of fLfs.

In the present study, we found that key enzymes that catalyze aerobic glycolysis are induced in human fLfs (hfLfs) isolated from IPF lungs or WT mouse fLfs (mfLfs) isolated from lungs of mice with established pulmonary fibrosis. We (22) and others (23, 24) have reported that CAV1 expression is markedly reduced in fLfs. Restoration of CAV1 expression or treatment with CAV1 scaffolding domain peptide (CSP) — or its 7–amino acid deletion fragment CSP7 — inhibits expression of profibrotic marker proteins in fLfs (22, 25). We further found that treatment of fLfs with CSP or CSP7 inhibited the increase in glycolysis in fLfs, which was associated with the antifibrogenic effects of these peptides. The process involves restoration of baseline p53 and its downstream transcriptional target, microRNA-34a (miR-34a) expression, which are otherwise reduced in hfLfs or mfLfs. Our study has established that CSP7-mediated restoration of baseline p53 and miR-34a mitigates fibrotic phenotypes by restraining augmented glycolysis in fLfs.

## Results

### Dysregulated glucose metabolism in fLfs.

**Figure 1:**
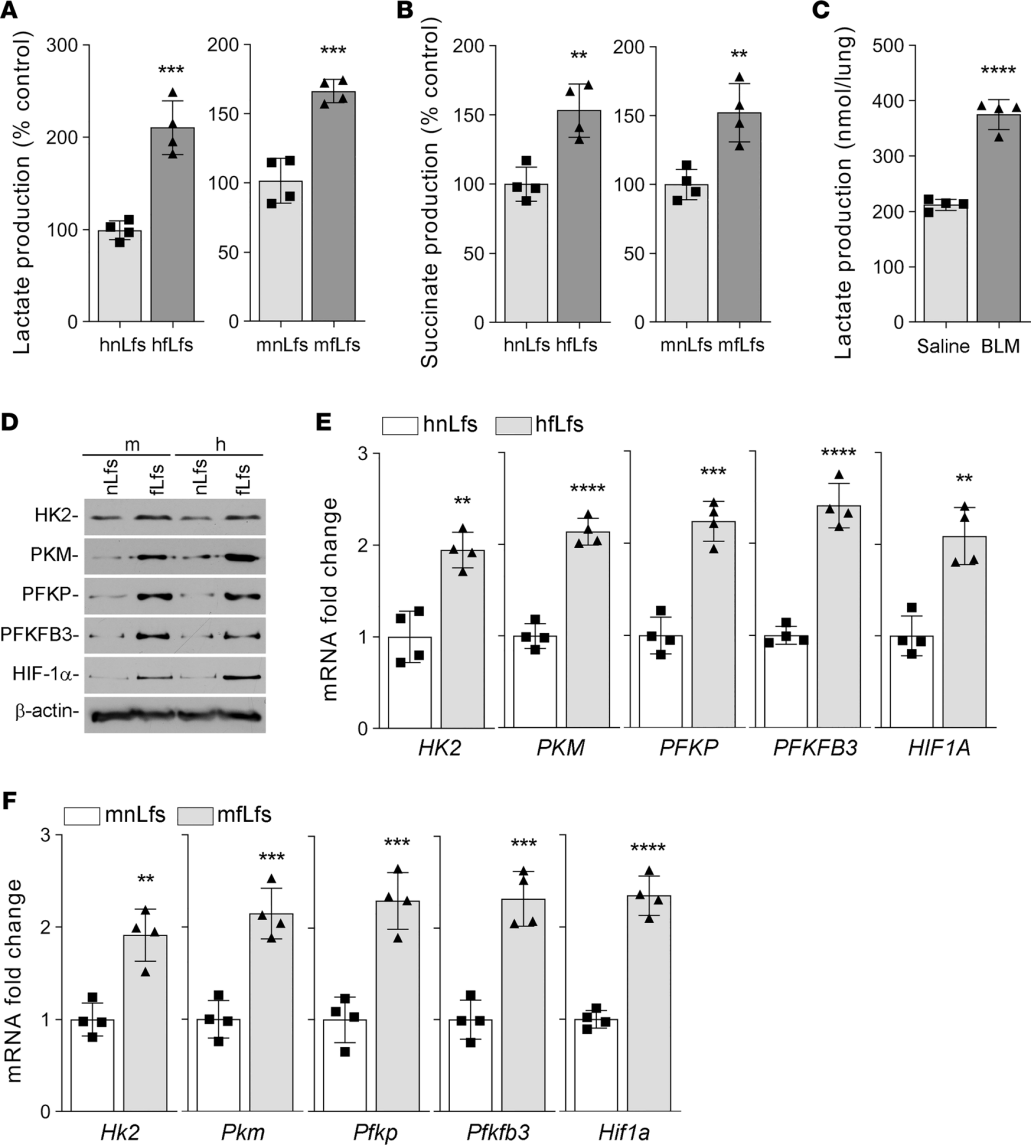
Augmented glycolysis in fLfs. (**A** and **B**) Primary human normal lung fibroblasts (hnLfs) isolated from the lungs of healthy donors (*n* = 4) and human fibrotic lung fibroblasts (hfLfs) from patients with IPF (*n* = 4), or mouse nLfs (mnLfs) from the lungs of mice without lung injury (*n* = 4) or mouse fLfs (mfLfs) from mice with BLM-induced established pulmonary fibrosis (*n* = 4) were measured for the cellular lactate (**A**) and succinate (**B**) levels. (**C**) Whole lung homogenates from mice without lung injury (*n* = 4) or with BLM-induced pulmonary fibrosis (*n* = 4) were analyzed for total lactate content. (**D**) Whole cell lysates from the mnLf and mfLf or hnLf and hfLf were analyzed for the differential expression of HK2, PKM, PFKP, PFKFB3, and HIF-1α protein by Western blotting. Images are representative of 2 independent experiments. (**E** and **F**) Total RNA isolated from hnLfs and hfLfs (**E**) or from mnLfs and mfLfs (**F**) as in **A** and **B** were tested for changes in relative *HK2*, *PKM*, *PFKP*, *PFKFB3*, and *HIF1A* mRNA expression levels by qPCR. Data pooled from 2 independent experiments are represented as mean ± SD analyzed by Student’s *t* test. ** *P* < 0.01, *** *P* < 0.001, **** *P* < 0.0001.

To determine the dysregulated glucose metabolism during mesenchymal cell dedifferentiation, normal lung fibroblasts (nLfs) — isolated from human donors without ILDs or mice without experimentally induced lung injury — and fLfs were tested for cellular lactate and succinate levels. We found that both lactate (Figure 1A) and succinate (Figure 1B) levels were significantly increased in hfLfs and mfLfs when compared with their expression in corresponding nLfs. To confirm that glycolytic reprogramming does occur in vivo, we measured lactate levels in lung tissues of mice with bleomycin-induced (BLM-induced) established pulmonary fibrosis. As shown in the Figure 1C, lactate levels were markedly increased in the fibrotic lung tissues of mice with BLM-induced lung injury, compared with lactate levels in normal lung tissues of mice without BLM lung injury. We assessed the relative expression of key enzymes that control glycolytic flux rate in murine and human nLfs and fLfs. We found that glycolytic enzymes such as hexokinase-2 (HK2), pyruvate kinase (PKM), phosphofructokinase (PFKP), and 6-phosphofructo-2-kinase/fructose-2,6-biphosphatase 3 (PFKFB3) levels were markedly upregulated in mfLfs and hfLfs (Figure 1D). These changes were associated with a parallel increase in hypoxia inducible factor-1α (HIF-1α), a key transcriptional factor that drives anaerobic glycolysis. Consistent with protein expression, quantitative PCR (qPCR) analyses using primers listed in Table 1 revealed a significant increase in the expression of their mRNAs in hfLfs (Figure 1E) and mfLfs (Figure 1F). These results clearly suggest altered glycolysis in differentiated fLfs.

### p53 regulates enzymes involved in glucose metabolism.

**Figure 2:**
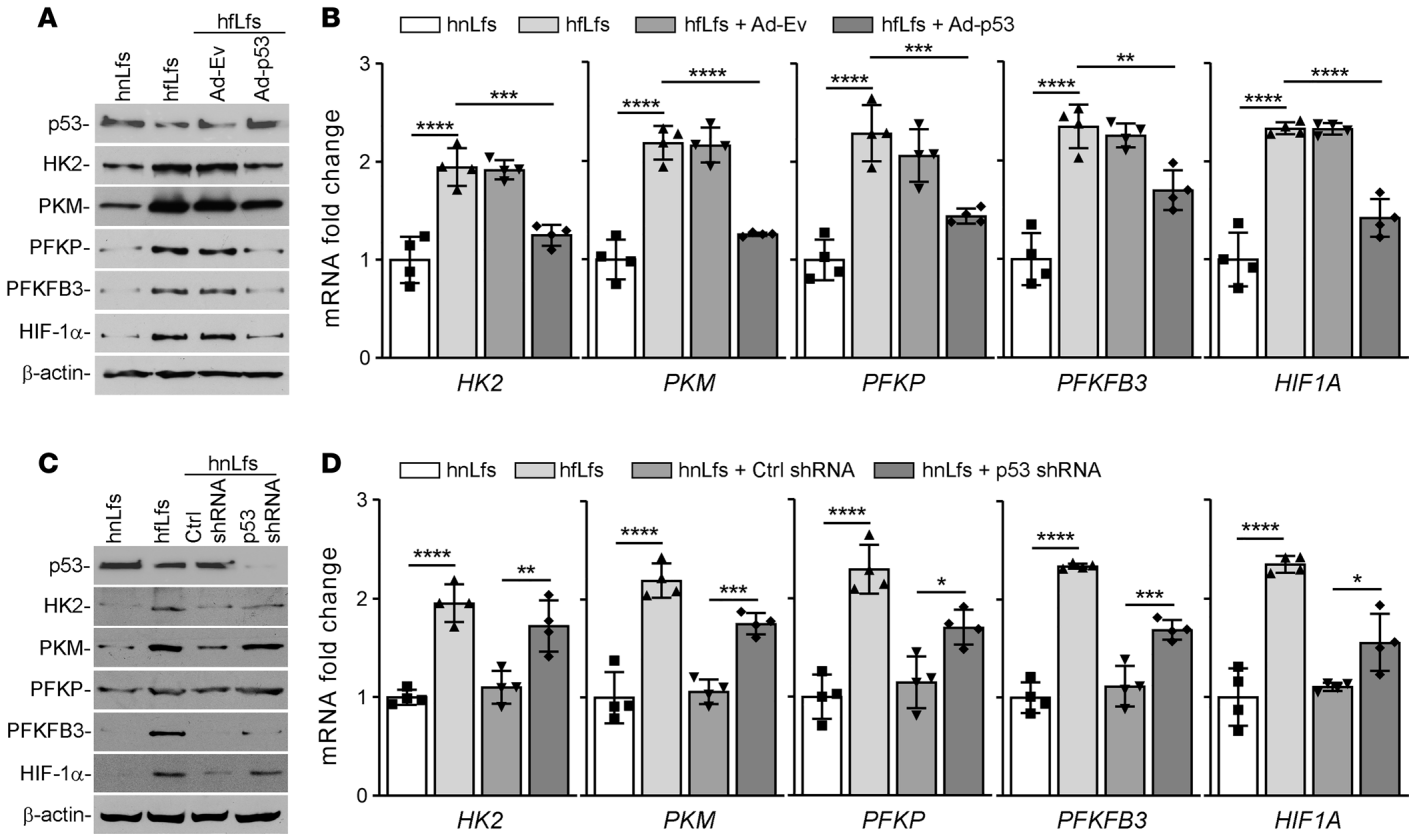
Regulation of glucose metabolism by p53 in fLfs. (**A**) hfLfs were transduced with adenoviral vector expressing empty vector (Ad-Ev) or p53 (Ad-p53). Naive hnLfs and hfLfs were used as controls. Forty-eight hours later, the cell lysates were immunoblotted for p53, HK2, PKM, PFKP, PFKFB3, and HIF-1α proteins. Images are representative of 2 independent experiments. (**B**) Total RNA isolated from *n* = 4 naive hnLf and hfLfs, or hfLfs transduced with Ad-Ev or Ad-p53 as in **A**, were analyzed for *HK2*, *PKM*, *PFKP*, *PFKFB3*, and *HIF1A* mRNA expression by qPCR (*n* = 4). (**C**) hnLfs were transduced with lentiviral vector–expressing control shRNA (Ctrl shRNA) or p53 shRNA. Naive hnLfs and hfLfs were used as controls. After 48 hours, the cell lysates were analyzed for the expression of p53 and glycolytic markers by Western blotting. Images are representative of 2 independent experiments. (**D**) Total RNA isolated from *n* = 4 naive hnLfs and hfLfs, or hnLfs transduced with Ctrl shRNA or p53 shRNA, were tested for their mRNA expression by qPCR. Data represented as mean ± SD were analyzed by 1-way ANOVA followed by Tukey’s post hoc test. *P < 0.05, **P < 0.01, ***P < 0.001, ****P < 0.0001.

To examine the role of *p53* regulation of glucose metabolism, primary fLfs isolated from the lungs of IPF patients or mice with existing pulmonary fibrosis were transduced with adenoviral vector–expressing p53 (Ad-p53) or adenoviral empty vector (Ad-Ev). Overexpression of p53 in hfLfs from IPF tissue explants reduced the expression of HK2, PKM, PFKP, PFKFB3, and HIF-1α, while naive fLfs or fLfs exposed to Ad-Ev still displayed elevated baseline levels of glycolytic enzymes (Figure 2A). Consistent with these findings, corresponding mRNA levels of these enzymes and *HIF1A* were decreased following forced expression of p53 in fLfs (Figure 2B). To confirm whether the blockade of p53 expression contributes to augmented glycolysis, we treated nLfs with lentiviral vector–expressing (Lv-expressing) p53 shRNA. Control nLfs were left untreated or exposed to Lv-expressing control shRNA (Ctrl shRNA). Inhibition of p53 expression in nLfs using specific shRNA augmented the expression of HK2, PKM, PFKP, PFKFB3, and HIF-1α, while nLfs exposed to Lv Ctrl shRNA failed to increase in the basal expression of these enzymes (Figure 2C). This was further confirmed at the mRNA levels (Figure 2D), demonstrating a link between loss of baseline p53 to glycolytic reprogramming in fLfs.

### miR-34a regulates glucose metabolism.

**Figure 3:**
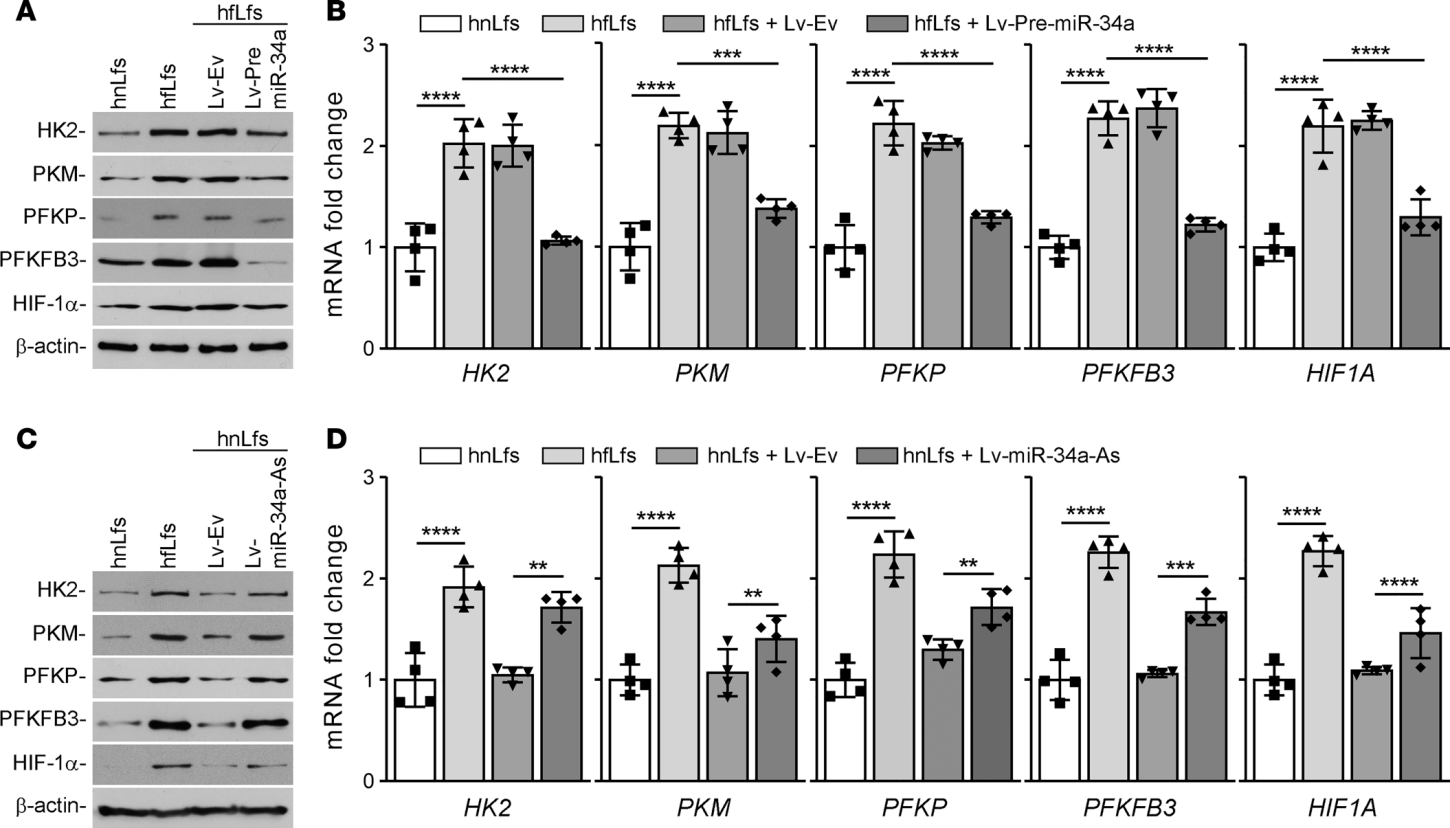
Regulation of glucose metabolism by miR-34a in fLfs. (**A**) hfLfs were transduced with lentivirus-expressing empty vector (Lv-Ev) or –precursor–miR-34a (Lv–Pre–miR-34a) to overexpress miR-34a. Naive hnLfs and hfLfs were used as controls. After 48 hours of infection, total cell extracts were analyzed for HK2, PFKP, PKM, PFKFB3, and HIF-1α by immunoblotting. Images are representative of 2 independent experiments. (**B**) Total RNA isolated from *n* = 4 naive hnLf and hfLfs, or hfLfs transduced with Lv-Ev or Lv–Pre–miR-34a as in **A**, was analyzed for *HK2*, *PFKP*, *PKM*, *PFKFB3*, and *HIF1A* by qPCR (*n* = 4). (**C**) hnLfs were transduced with Lv-Ev or Lv-expressing miR-34a antisense (Lv–miR-34a–As). Naive hnLfs and hfLfs were used as controls. After 48 hours of infection, the cell lysates were analyzed for the expression of HK2, PFKP, PKM, PFKFB3, and HIF-1α by Western blotting. The representative images of 2 independent experiments are shown. (**D**) Total RNA isolated from *n* = 4 naive hnLfs or hfLfs, or hnLfs transduced with Lv-Ev or Lv–miR-34a–As as in **C**, was tested for *HK2*, *PFKP*, *PKM*, *PFKFB3*, and *HIF1A* mRNA by qPCR (*n* = 4). Data represented as mean ± SD were analyzed by 1-way ANOVA followed by Tukey’s post hoc test. **P < 0.01, ***P < 0.001, ****P < 0.0001.

Activation of p53 induces various noncoding miRNAs, particularly miR-34a. Thus, to elucidate the possible role of miR-34a in regulating glucose metabolism, hfLfs were transduced with Lv-expressing precursor miR-34a (Lv–Pre–miR-34a) or Lv-Ev. *HK2*, *PFKP*, *PKM*, *PFKFB3*, and *HIF1A* transcriptions were repressed when hfLfs were infected with the Lv-expressing miR-34a, which led to a decrease in protein levels (Figure 3A). hfLfs infected with Lv-Ev showed no significant difference in either mRNA (Figure 3B) and protein level when compared with the naive cells. To further investigate whether reduced expression of these enzymes is directly related to restoration of miR-34a, we treated human nLfs with Lv-expressing miR-34a antisense (Lv–miR-34a–As). We found that inhibition of miR-34a augmented the levels of HK2, PFKP, PKM, PFKFB3, and HIF-1α protein (Figure 3C) and their mRNAs (Figure 3D). This indicates that miR-34a directly regulates the expression of enzymes involved in glucose metabolism.

### CSP and CSP7 regulate glycolysis in fLfs.

**Figure 4:**
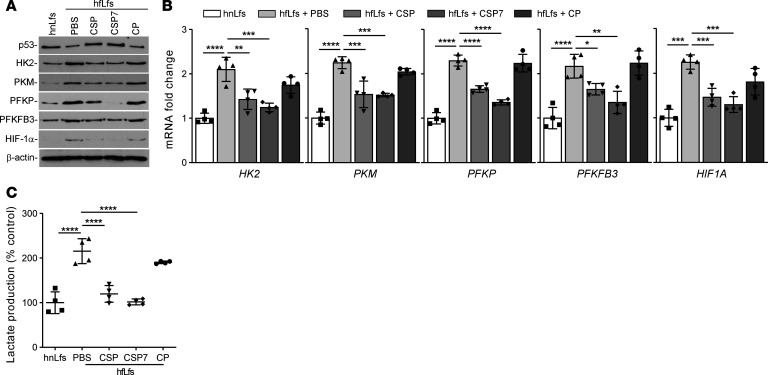
CSP and CSP7 inhibited augmented glycolysis in fLfs. (**A**) hfLfs were treated with or without 10 μM of CSP, CSP7, or CP for 48 hours. The cell lysates were immunoblotted for the differential expression of p53, HK2, PKM, PFKP, PFKFB3, and HIF-1α proteins. The experiments were repeated twice. (**B**) Total RNA isolated from hnLfs, and hfLfs treated with or without CSP, CSP7, or CP as in **A**, were analyzed by qPCR for changes in mRNA expression of above listed glycolytic enzymes. Data pooled from 2 independent experiments (*n* = 4) are represented as mean ± SD. (**C**) The changes in the cellular lactate contents were measured in untreated hnLfs and hfLfs, as well as hfLfs treated with CSP, CSP7, and CP. Data pooled from 2 independent experiments (*n* = 4) are represented as mean ± SD. *P < 0.05, **P < 0.01, ***P < 0.001, ****P < 0.001, by 1-way ANOVA followed by Tukey’s post hoc test.

We recently demonstrated that CSP or its deletion fragment CSP7 exerts antifibrotic responses through restoration of baseline p53 and PAI-1 expression in fLfs (22, 25). To test whether this process involves changes in glycolytic flux, hfLfs were treated with CSP or CSP7 or control peptide of scrambled sequence (CP) as we described earlier (22). As shown in the Figure 4A, treatment of hfLfs with either CSP or CSP7 has caused a marked reduction in the expression of HK2, PKM, PFKP, PFKFB3, and HIF-1α. These changes were associated with parallel increase in basal p53 expression in hfLfs. Naive hfLfs and hfLfs treated with CP still showed elevated levels of glycolytic enzymes and low baseline p53. Consistent with protein expression, we also found significant reduction in the expression of *HK2*, *PKM*, *PFKP*, *PFKFB3*, and *HIF1A* mRNA in hfLfs treated with CSP or CSP7, while those exposed to CP failed to respond to the treatment (Figure 4B). These changes were further reflected with significant reduction in otherwise elevated cellular lactate levels in hfLfs treated with CSP or CSP7 when compared with those exposed to CP (Figure 4C).

**Figure 5:**
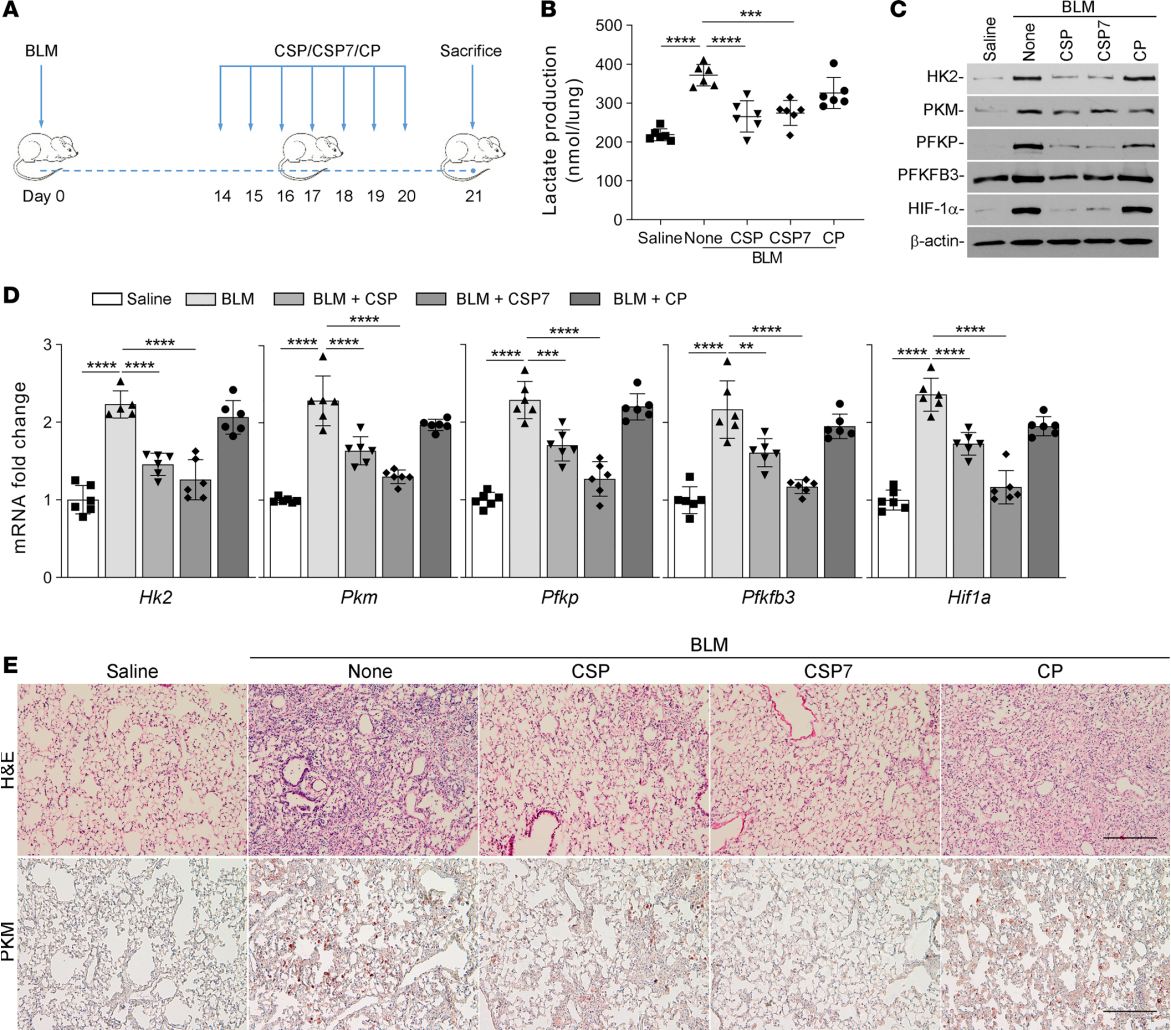
CSP7 attenuates augmented glycolysis in WT mice with BLM-induced pulmonary fibrosis. (**A**) Schematic illustration of the experimental design. WT mice were exposed once to BLM (8 U/kg in 50 μL saline) through intranasal instillation to cause established pulmonary fibrosis. Two weeks after BLM exposure, mice were treated with 1.5 mg/kg of CSP, CSP7, or CP via i.p. injection daily for 7 consecutive days. All mice were euthanized 21 days after BLM exposure. (**B**) The lungs were harvested, and lung homogenates were tested for total lactate. (**C**) Total lung homogenates were evaluated for the changes in the expression of HK2, PKM, PFKP, PFKFB3, and HIF-1α proteins by Western blotting. The experiments were repeated 2 times. (**D**) Total mouse lung RNA was analyzed for *Hk2*, *Pkm*, *Pfkp*, *Pfkfb3*, and *Hif1a* mRNA by qPCR. Data pooled from 2 independent experiments (*n* = 6 per group) are represented as mean ± SD. **P < 0.01, ***P < 0.001, ****P < 0.0001 were obtained by 1-way ANOVA followed by Tukey’s post hoc test. (**E**) The lung sections were subjected to histological analysis, and representative images of H&E and IHC staining for PKM are shown. Scale bars: 200 μm.

*CSP or CSP7 attenuates elevated glucose metabolism in mice with single-dose (1*×*) BLM-induced pulmonary fibrosis*. We recently reported that both CSP or CSP7 treatment resolves existing pulmonary fibrosis in WT mice (22, 25). To test whether these antifibrogenic peptides can restore dysregulated glucose metabolism in mice with lung fibrosis, we started CSP or CSP7 treatment by daily i.p. injection 2 weeks after intranasal instillation of BLM as shown in Figure 5A. Treatment of mice having BLM-induced established pulmonary fibrosis with either CSP or CSP7 resulted in a significant reduction in total lung lactate levels, suggesting reversal of augmented glycolysis observed in mice with BLM-induced pulmonary fibrosis (Figure 5B). Total lung homogenates of WT mice exposed to BLM showed elevated expression of glycolytic markers. Mice treated with CSP or CSP7 exhibited a marked reduction in the expression of screened glycolytic enzymes, along with restoration of basal p53 expression (Figure 5C) and attenuation of BLM-induced lung fibrosis (22, 25). Consistent with protein expression, we also found that the expression of *Hk2*, *Pkm*, *Pfkp*, *Pfkfb3*, and *Hif1a* mRNA was significantly elevated in the lungs of mice treated with BLM. This was significantly reduced in these mice following treatment with CSP or CSP7. However, those treated with CP still showed elevated mRNA expression (Figure 5D). In accordance with our earlier observations (22, 25), H&E staining of lung sections indicated that mice exposed to BLM developed pulmonary fibrosis, which was markedly reduced in mice exposed to CSP or CSP7. Consistent with the H&E staining pattern, IHC analysis confirmed reduced staining for PKM antigen in lung sections of mice following peptide treatment (Figure 5E). However, those exposed to CP still showed increased PKM antigen levels.

### Inhibition of augmented glucose metabolism in mice with TGF-β1–induced lung fibrosis by CSP or CSP7 in vivo.

**Figure 6:**
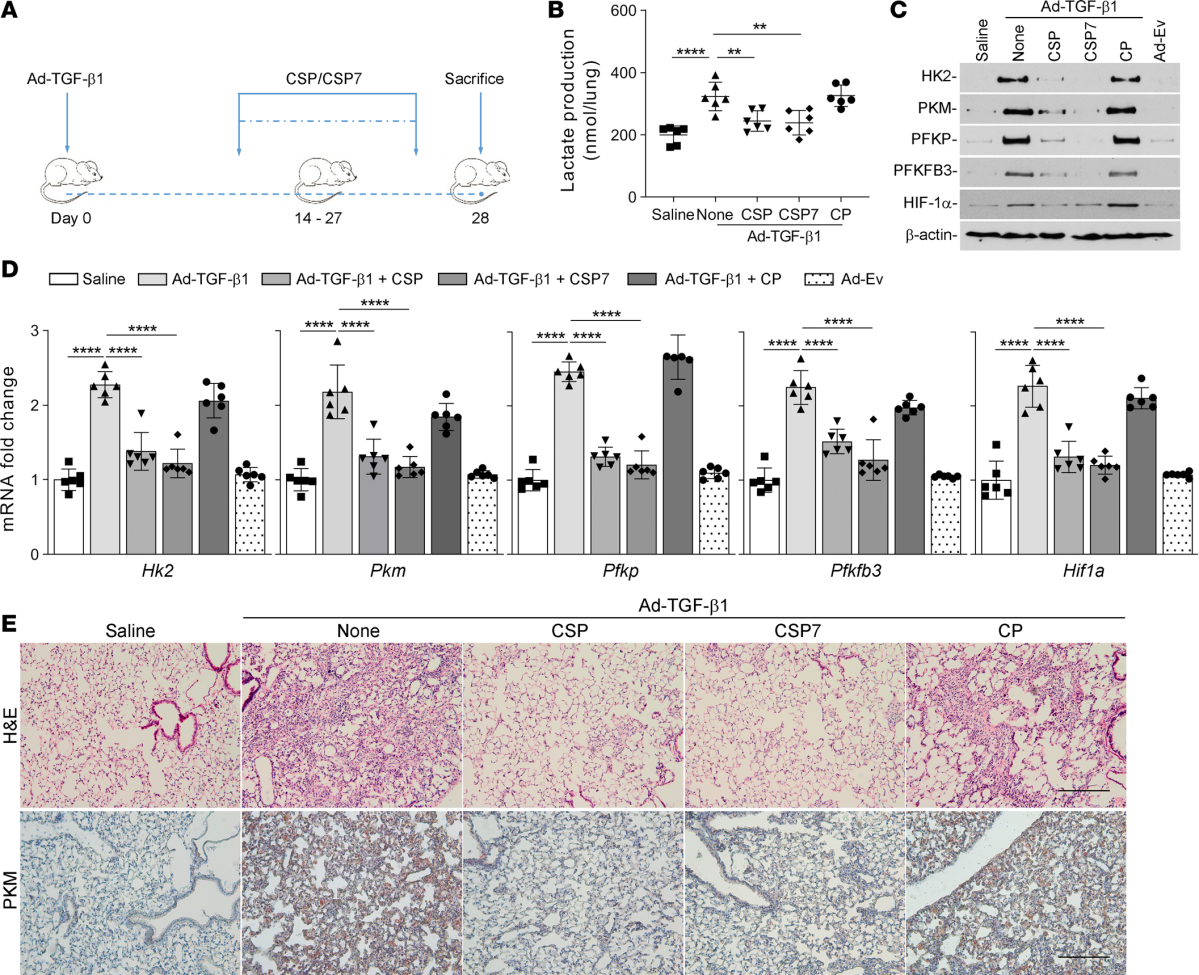
CSP7 restores dysregulated glucose metabolism in WT mice with TGF-β1–induced pulmonary fibrosis. (**A**) Schematic illustration of the experimental design. WT mice were transduced with 1 × 10^9^ pfu of Ad vector–expressing constitutively active TGF-β1 (Ad–TGF-β1). Two weeks after exposure, mice were i.p. injected with or without 1.5 mg/kg of CSP7 or CP for 14 days. At the end of treatment, mice were euthanized. (**B**) Lung homogenates were analyzed for total lactate contents. (**C**) Lung homogenates were immunoblotted for the differential expression of HK2, PKM, PFKP, PFKFB3, and HIF-1α protein. Representative images from 2 independent experiments are shown. (**D**) Total lung *Hk2*, *Pkm*, *Pfkp*, *Pfkfb3*, and *Hif1a* mRNA levels were quantified by qPCR. The experiments were repeated 2 times. Data pooled from 2 independent experiments (*n* = 6 per group) are represented as mean ± SD. **P < 0.01, ***P < 0.001, ****P < 0.0001 by 1-way ANOVA followed by Tukey’s post hoc test. (**E**) The lung sections were subjected to histological analysis, and representative images of H&E and IHC staining for PKM are shown. Scale bars: 200 μm.

To further test the effect of CSP or CSP7 on glucose metabolism, mice with adenoviral vector–expressing constitutively active TGF-β1–induced (Ad–TGF-β1–induced) established pulmonary fibrosis were daily exposed to CSP or CSP7 by i.p. injection for 2 weeks starting 14 days after Ad–TGF-β1 transduction (Figure 6A). As shown in Figure 6B, treatment of mice having existing lung fibrosis with either CSP or CSP7 significantly reduced total lactate levels in lung tissues. However, this did not occur in mice with Ad–TGF-β1–induced lung fibrosis left untreated or exposed to CP. The changes in total lactate levels were associated with marked reduction in Ad–TGF-β1–induced pulmonary collagen deposition in mice exposed to CSP or CSP7, compared with that of CP-treated mice (25), indicating a causal link between dysregulated glycolysis and profibrogenic remodeling. Immunoblotting of total lung homogenates from WT mice exposed to Ad–TGF-β1 showed a striking increase in the expression of HK2, PKM, PFKP, PFKFB3, and HIF-1α. These changes were remarkably reduced in WT mice harboring lung fibrosis and treated with CSP or CSP7, illustrating reversal of glycolytic reprogramming and dedifferentiation of fLfs (Figure 6C). In agreement with protein expression, qPCR analysis of whole lung total RNA of Ad–TGF-β1–treated mice showed a significant increase in the expression of *Hk2*, *Pkm*, *Pfkp*, *Pfkfb3*, and *Hif1a* transcripts compared with the corresponding levels in untreated or Ad-Ev–treated control mice. However, these changes were significantly suppressed to baseline levels in WT mice exposed to Ad–TGF-β1 and later treated with CSP or CSP7, while their expression levels remained elevated in mice treated with CP (Figure 6D). H&E staining of lung sections indicated Ad–TGF-β1–induced pulmonary fibrosis, which was markedly reduced in those treated with CSP or CSP7 (Figure 6E). IHC analysis further confirmed the inhibition of the otherwise BLM-induced increase in PKM antigen staining in the lung tissues of mice after treatment with either CSP or CSP7. However, elevated staining for PKM was observed in lung sections of WT mice exposed to Ad–TGF-β1 and treated with CP.

### Mitigation of multihit (8×) BLM-induced aberration in glycolysis by CSP or CSP7 in vivo.

**Figure 7:**
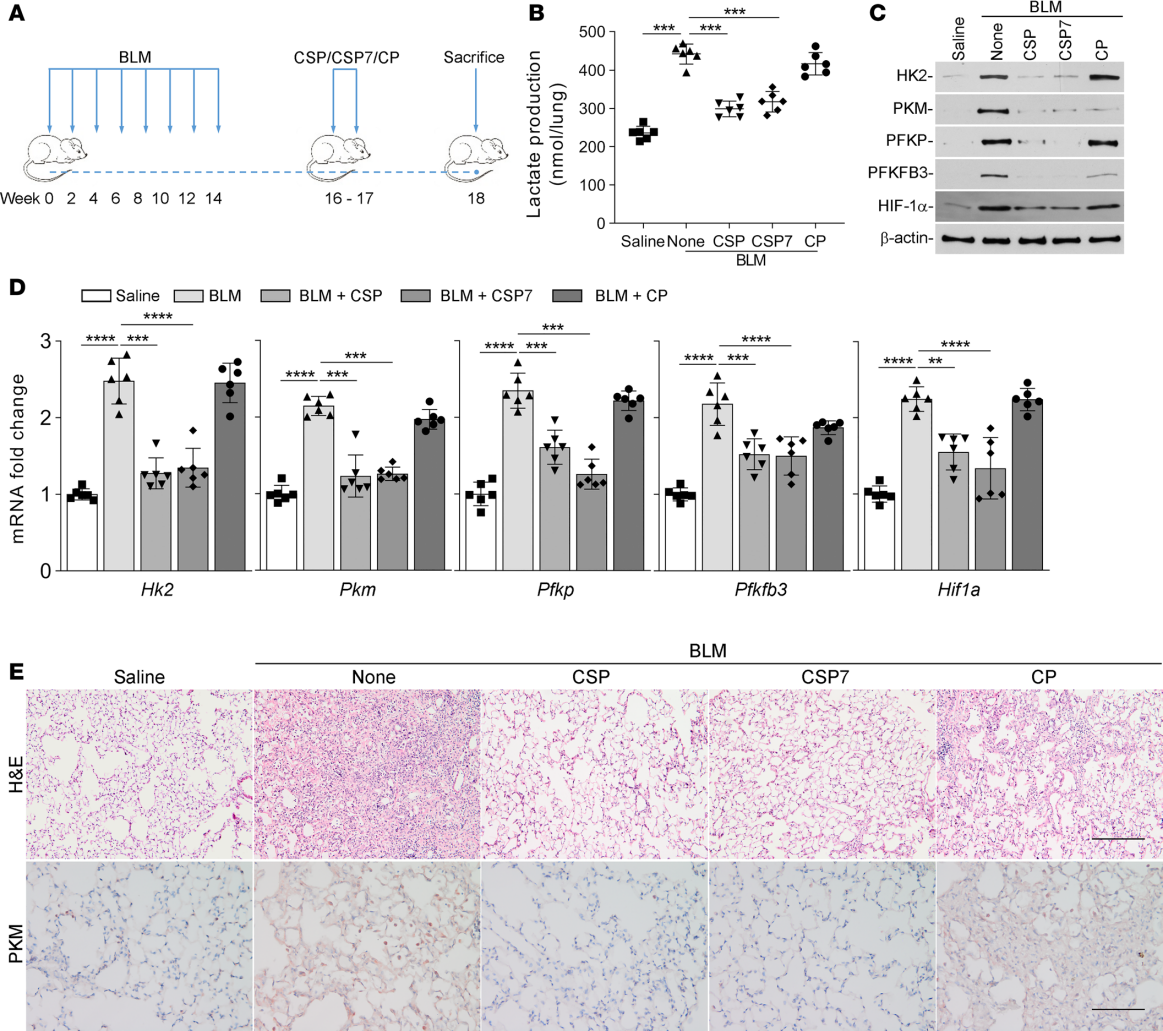
CSP7 mitigates enhanced glucose metabolism in WT mice with multiple-dose BLM-induced pulmonary fibrosis. (**A**) Schematic illustration of the experimental design. WT mice were exposed to BLM (2 U/kg in 50 μL saline) once every 2 weeks for a total of 8 times in 4 months by intranasal instillation under anesthesia to induce pulmonary fibrosis. Two weeks after the final dose of BLM exposure, mice with BLM-induced pulmonary fibrosis were exposed to 1.5 mg/kg of CSP, CSP7, and CP by i.p. injection daily for 2 weeks. (**B**) At the end of treatment, mice were euthanized, and whole lung homogenates were analyzed for total lactate contents. (**C**) Total protein from the whole lung homogenates were immunoblotted for HK2, PKM, PFKP, PFKFB3, and HIF-1α protein. The experiments were repeated 2 times. (**D**) Total lung RNA was subjected to qPCR for changes in the expression of *Hk2*, *Pkm*, *Pfkp*, *Pfkfb3*, and *Hif1a* mRNA. Data pooled from 2 independent experiments (*n* = 6 per group) are represented as mean ± SD. **P < 0.01, ****P < 0.0001 by 1-way ANOVA followed by Tukey’s post hoc test. (**E**) The lung sections were subjected to histological chemical analysis, and representative images of H&E and IHC staining for PKM are shown. Scale bars: 200 μm.

To further test the glycolytic regulatory effect of CSP or CSP7, we performed a similar experiment with the 8× BLM–induced established pulmonary fibrosis model. CSP or CSP7 was given daily by i.p. injection for 2 weeks after the eighth dosage of BLM (Figure 7A). As shown in Figure 7B, CSP or CSP7 reduced BLM-induced total lung lactate levels, while those exposed to CP remained unchanged. These changes were associated with a significant reduction in BLM-induced whole lung collagen content in WT mice exposed to CSP or CSP7 and not in those treated with CP (22, 25), indicating resolution of existing pulmonary fibrosis. Further analysis of total lung homogenates of WT mice exposed to 8× BLM showed increased expression of HK2, PKM, PFKP, PFKFB3, and HIF-1α, which was consistent with changes observed in mice treated with single dose BLM and Ad–TGF-β1. Further, reversal of 8× BLM–induced changes was observed in mice treated with CSP or CSP7, restored near normal baseline expression of the glycolytic enzymes (Figure 7C), along with attenuation of BLM-induced lung fibrosis (22, 25). Consistent with protein expression, we also found that *Hk2*, *Pkm*, *Pfkp*, and *Pfkfb3* mRNA were similarly elevated in the lungs of these mice. This was significantly reversed in mice exposed to BLM and treated with CSP or CSP7, while those treated with CP still showed elevated mRNA expression (Figure 7D). H&E staining of lung sections indicated BLM-induced pulmonary fibrosis, which was markedly reduced in those treated with either CSP or CSP7 (Figure 7E). IHC analysis further confirmed the inhibition of the otherwise BLM-induced increase in PKM antigen staining in the lung tissues of mice after CSP or CSP7 treatment. However, elevated staining for PKM antigen was apparent in lung sections of mice treated with CP after causing BLM-induced pulmonary fibrosis.

### Inhibition of p53 abrogates CSP- and CSP7-mediated restoration of glycolytic flux rate.

**Figure 8:**
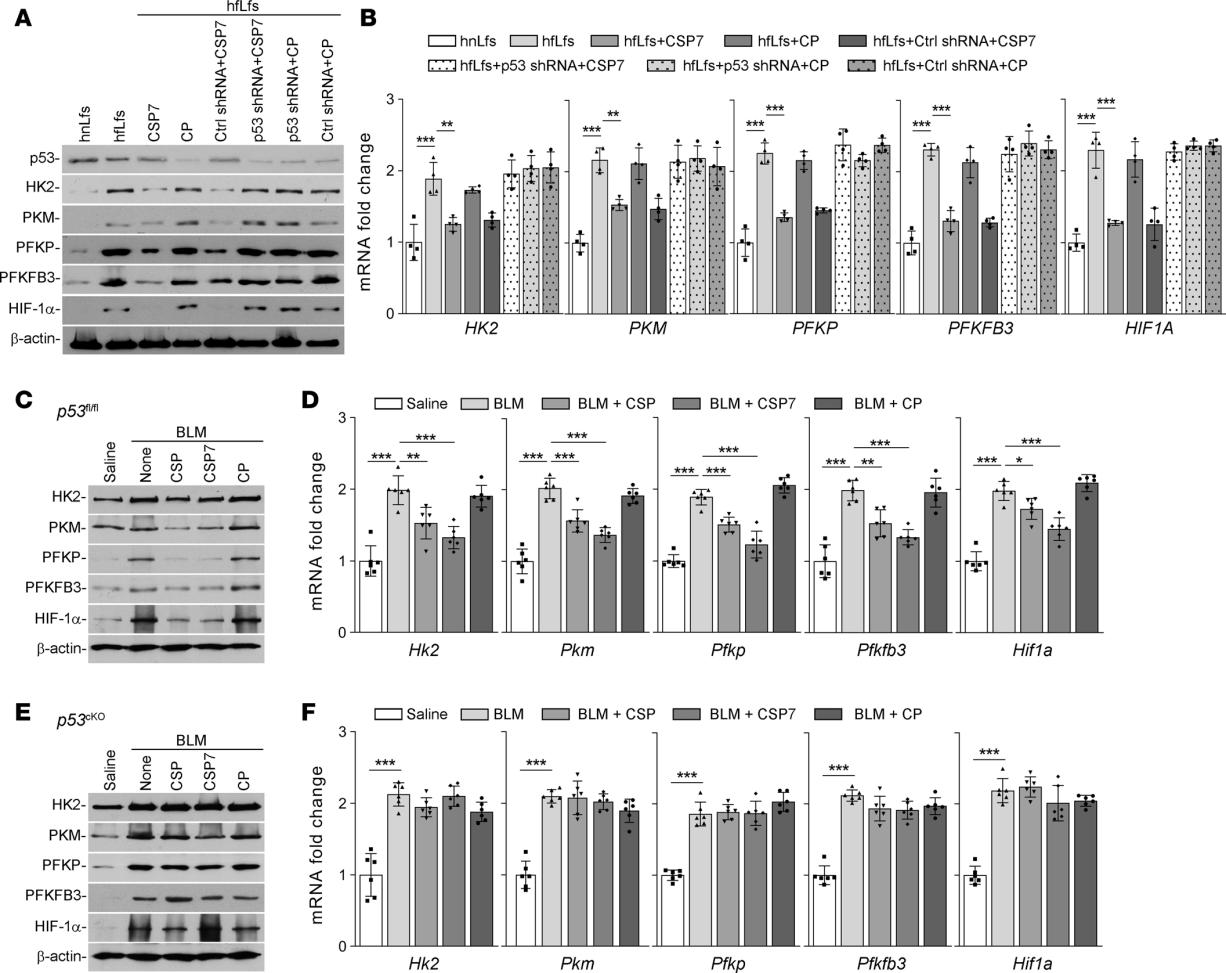
Inhibition of *p53* abrogates the ability CSP or CSP7 to restore glycolysis in lungs. hfLfs transduced with or without Lv-p53-shRNA or Ctrl shRNA were treated with PBS, CSP7, or CP for 48 hours. Naive hnLfs and hfLfs were used as controls. (**A**) Cell lysates were immunoblotted for the differential expression of p53, HK2, PKM, PFKP, PFKFB3, and HIF-1α proteins. The experiments were repeated twice. (**B**) Total RNA isolated from *n* = 4 hnLfs, and hfLfs treated as in **A**, were quantified by qPCR for above mentioned mRNA. *p53^fl/fl^* and tamoxifen inducible *p53*^cKO^ mice (*n* = 6 per group) were exposed to saline or BLM. Two weeks after BLM injury, mice were treated with or without CSP, CSP7, or CP by i.p. injection daily for 7 consecutive days. (**C** and **E**) At the end of the treatment, mice were euthanized, and the lung homogenates from *p53^fl/fl^* (**C**) and *p53*^cKO^ (**E**) mice were immunoblotted for the differential expression of marker enzymes involved in glycolysis. The experiments were repeated 2 times. (**D** and **F**) Total lung RNA from *p53^fl/fl^* (**D**) and *p53*^cKO^ (**F**) mice were analyzed for *Hk2*, *Pkm*, *Pfkp*, *Pfkfb3*, and *Hif1a* mRNA by qPCR. Data pooled from 2 independent experiments are represented as mean ± SD and analyzed by 1-way ANOVA followed by Tukey’s post hoc test. *P < 0.05, **P < 0.01, ***P < 0.001.

To confirm that the restoration of p53 expression is required for the reversal of dysregulated glycolysis by CSP or CSP7 in hfLfs, we transduced fLfs with p53 shRNA and later treated these fLf lines with either CSP7 or CP. The responses were compared with naive hfLfs treated with CSP7 or CP, or hfLfs transduced with Ctrl shRNA and treated with CSP7 or CP. As showed in Figure 8A, CSP7 treatment reduced expression of glycolytic markers in naive hfLfs or fLfs transduced with Ctrl shRNA, indicating its beneficial effects in fLfs. These changes were associated with elevation of basal p53 levels by CSP7 treatment. Interestingly, these effects were not found in fLfs treated with p53 shRNA. This was further confirmed at the *HK2*, *PFKP*, *PKM*, *PFKFB3*, and *HIF1A* mRNA levels (Figure 8B). These findings were consistent with lack of suppression of profibrogenic markers, proliferation, migration, and invasion by CSP or CSP7 in fLfs treated with p53 shRNA (22).

To further confirm the involvement of p53 in regulation of glycolysis in fLfs in vivo, tamoxifen inducible conditional KO mice lacking p53 expression in fibroblasts (*p53*^cKO^ mice) were exposed to BLM, and 14 days later, these mice were exposed to vehicle, CSP, CSP7, or CP. *p53^fl/fl^* mice treated with CSP or CSP7 under same condition were used as controls. Pulmonary fibrosis induced with BLM caused dysregulation in glycolysis in both *p53^fl/fl^* (Figure 8, C and D) and *p53*^cKO^ (Figure 8, E and F) mice, as indicated by the increased expression of HK2, PKM, PFKP, PFKFB3, and HIF-1α protein and their mRNAs in whole lung homogenates. Interestingly, neither CSP nor its deletion fragment CSP7 reduced BLM-induced increases in the expression of glycolytic enzymes in *p53*^cKO^. However, *p53^fl/fl^* mice exposed to either CSP or CSP7 showed remarkable suppression of glycolytic pathway markers. These changes were associated with concurrent restoration of baseline p53 level, which is otherwise suppressed due to BLM-induced pulmonary fibrosis. Further analysis of total lung RNA for glycolytic marker mRNA confirmed that both CSP and CSP7 were effective against altered metabolism in *p53^fl/fl^* mice. This is in conformity with the resistance of *p53*^cKO^ and having pulmonary fibrosis to CSP7 or CSP7 treatment (22). The inability of CSP or CSP7 to suppress augmented glycolysis in BLM treated *p53*^cKO^ mice validates that CSP7-mediated restoration of baseline expression of p53 is required for resolution of existing lung fibrosis through the regulation of glucose metabolism.

### Inhibition of miR-34a abrogates regulatory effects of CSP7 in vitro and in vivo.

**Figure 9:**
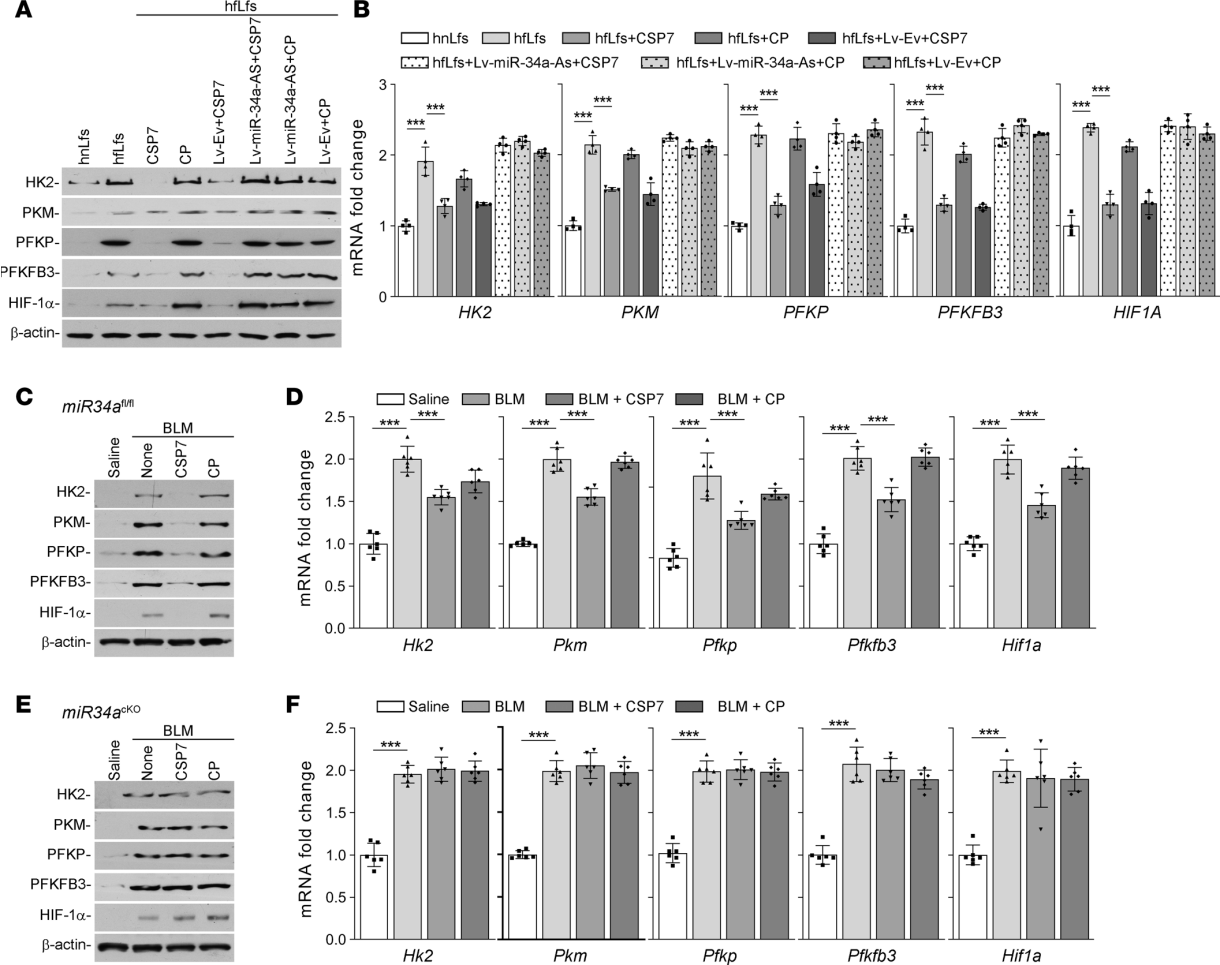
miR-34a inhibition revokes regulatory effect of CSP7. hfLfs transduced with Lv–miR-34a–As or Lv-Ev were treated with PBS, CSP7, or CP for 48 hours. Naive hnLfs and hfLfs were used as controls. (**A**) Cell lysates were immunoblotted for the differential expression of HK2, PFKP, PKM, PFKFB3, and HIF-1α. The experiments were repeated 2 times. (**B**) mRNA expression levels were quantified by qPCR (*n* = 4). *miR34a^fl/fl^* and tamoxifen inducible *miR34a*^cKO^ mice (*n* = 6 per group) were exposed to saline or BLM. Two weeks after BLM injury, mice were i.p. injected with or without CSP7 or CP daily for 7 consecutive days. (**C** and **E**) At the end of the treatment, mice were euthanized and the lung homogenate of *miR34a^fl/fl^* (**C**) and *miR34a*^cKO^ (**E**) were immunoblotted for the differential expression of glycolytic markers. The experiments were repeated twice. (**D** and **F**) mRNA expression levels of mentioned genes from *miR34a^fl/fl^* (**D**) and *miR34a*^cKO^ (**F**) mice were quantified by qPCR. Data pooled from 2 independent experiments are represented as mean ± SD and analyzed by 1-way ANOVA followed by Tukey’s post hoc test. ***P < 0.001.

To confirm the involvement of miR-34a in the regulation of altered glucose metabolism by CSP7, we transduced hfLfs with Lv–miR-34a–As and later treated these cells with CSP7 or CP. The responses were compared with naive hfLfs or hfLfs transduced with Lv-Ev and treated with CSP7 or CP. Treatment with CSP7 caused marked reduction in the expression of glycolytic markers, indicating its regulatory effect in naive fLfs. These changes were not found in fLfs transduced with Lv–miR-34a–As (Figure 9A). This was further reflected at the levels of *HK2*, *PFKP*, *PKM*, *PFKFB3*, and *HIF1A* mRNA expression (Figure 9B).

*miR34a*^fl/fl^*Col*^Cre^ mice, generated by cross-breeding *miR34a^fl/fl^* mice with *Col1*^Cre^ mice, were i.p. injected with tamoxifen to suppress expression of miR-34a in fibroblasts through induction of Cre-recombinase. Tamoxifen inducible *miR34a^cKO^* mice, lacking miR-34a expression in fibroblasts, were exposed to BLM, and 14 days later, these mice were exposed to vehicle, CSP, CSP7, or CP. *miR34a^fl/fl^* mice under the same treatment conditions were used as controls. *miR34a^fl/fl^* and *miR34a*^cKO^ mice showed increased expression of HK2, PFKP, PKM, PFKFB3, and HIF-1α in whole lung homogenates 21 days after BLM lung injury. As expected, *miR34a^fl/fl^* mice responded to peptide treatment (Figure 9, C and D), whereas — in *miR34a*^cKO^ mice — CSP7 treatment did not reduce BLM-induced augmented glycolytic enzymes expression (Figure 9, E and F). This indicates that CSP7-mediated restoration of miR-34a downregulates the augmented glucose metabolism in fLfs. The inability of CSP7 to resolve augmented glycolysis in BLM-treated *p53*^cKO^ or *miR34a*^cKO^ mice establishes the importance of baseline p53 and miR-34a feedback induction in regulating fLfs glucose metabolism.

## Discussion

Dysregulated metabolic changes have been involved in the pathogenesis of many diseases, including IPF. Altered glucose metabolism is one of the major distinguishing phenotypes between normal and cancer cells (4, 5, 15). The essential role of glycolytic enzymes like HK2 and PFKFB3 in maintaining the high glycolytic tumor phenotypes has been reported in recent years. Studies suggest that activation of HIF-1α stimulates the expression of various glycolytic enzymes, which drives aerobic glycolysis for energy production (26). The mechanism contributing to glycolytic reprograming in fibrotic lungs is not well established, and the metabolism-based therapeutics for treating fibrotic lung disorders are lacking. In the current study, we have shown that glycolysis is increased in fLfs isolated from the fibrotic lung explants derived from patients with IPF, or from multiple murine models of existing pulmonary fibrosis. We further demonstrated that the process involves loss of baseline p53 expression and its inducible downstream target, miR-34a, in fLfs of IPF lungs and mice with established lung fibrosis.

Aberrant glycolysis in fibrotic lungs was measured by the production of lactate and succinate — the end products of glycolysis and the TCA cycle intermediate, respectively. These were found to be elevated in fLfs compared with their basal levels of expression found in nLfs. This was further confirmed by an approximate 2-fold increase in lactate content in fibrotic lungs of mice in vivo. Screening for the expression of key rate-limiting enzymes suggested upregulation of HK2, PKM, PFKP, PFKFB3, and HIF-1α in both hfLf and mfLf lines. A recent report from our group (22) demonstrated that loss of expression of p53 in fLfs of IPF lungs and mice with established pulmonary fibrosis contributes to their differentiation. The sequel of p53 activation induces several target genes, including *miR34a* (27). miR-34a in turn inhibits translation of histone deacetylase SIRT1 by directly binding its seed sequences present in 3′ untranslated region (28). This results in increased acetylation and stabilization of p53 for resistance of acetylated p53 from degradation by MDM2 (29), suggesting that activation of p53 and p53-mediated downstream induction of miR-34a through a feedback mechanism are essential steps in the regulation of fibroblasts phonotype and lung fibrosis. Consistent with these observations, Cui and colleagues (30) have reported that miR-34a inhibits pulmonary fibrosis through induction of senescence in lung fibroblasts. We therefore examined whether expression of p53 and/or miR-34a regulates the suppression of glycolysis in fLfs and found that inhibition of either p53 or miR-34a in nLfs contributed to glycolytic upregulation. On the contrary, p53 and/or miR-34a overexpression in fLfs reduced the expression of glycolytic marker enzymes, suggesting glycolytic suppression. Like most cancer cells, fLfs rely on glycolysis, which ultimately produces lactate and 2 adenosine triphosphate (ATP) molecules from a single molecule of glucose. Glycolysis is less energy efficient than oxidative phosphorylation, which generates 36 ATPs per 1 glucose molecule. However, glycolysis can generate ATP faster than mitochondrial oxidative phosphorylation (31). In addition, glycolysis may provide intermediaries that are necessary for biosynthesis of amino acids, lipids, nucleotides, and lactate to maintain redox balance. Thus, glycolytic upregulation could confer survival benefits to fLfs, as seen in cancer cells (32). The importance of glycolysis in the pathogenesis of progressive fibrosis is further supported by the recent reports demonstrating pharmacological intervention that targets cellular metabolism, such as metformin or other activators of AMP-activated protein kinase (AMPK) mitigate pulmonary fibrosis through deactivation of myofibroblasts (15, 21, 33, 34). Furthermore, AMPK acts as an upstream activator of p53 and p53 transactivates Sestrin1 and Sestrin2, resulting AMP phosphorylation, which inhibits mTOR activity (35).

A principal component of caveolae, CAV1, interacts with signaling proteins via CAV1 scaffolding domain (36). CAV1 has been reported in the regulation of numerous signaling pathways and biological processes. CAV1 expression is reduced in fLfs in fibrotic lung disease (24). Multiple mechanisms may be involved in p53 regulation. As a binding protein for MDM2, CAV1 stabilizes p53 via sequestering MDM2 away from p53. CAV1 activates p53 via inhibiting SIRT1 (37), and elevated basal expression of SIRT1 has been observed in fLfs (38), probably due to loss of baseline p53 and miR-34a feedback induction in these cells. Furthermore, loss of CAV1 in fLfs results in low membrane–associated phosphatase and tensin homolog (PTEN) expression leading to aberrant activation the PI3K/AKT signaling pathway (39). These changes could further enhance MDM2-mediated ubiquitination and degradation of p53 protein (40). We recently reported that expression of both CAV1 and p53 protein are markedly reduced in fLfs, and forced expression of CAV1 or CSP/CSP7 restored basal p53 and reduced profibrogenic proteins in fLfs (22, 25). Interestingly, CSP also inhibits BLM- or silica-induced lung injury and prevents development of pulmonary fibrosis by blocking otherwise increased p53 and miR-34a by competing with increased CAV1 expression in injured alveolar epithelial cells (41–43). In this study, we found that CSP or CSP7 attenuated the augmented glycolysis by inducing the expression of p53 and miR-34a, which are otherwise reduced in fLfs isolated from fibrotic lungs of patients with IPF or mice with BLM-induced established pulmonary fibrosis. This is consistent with resolution of existing lung fibrosis by CSP and its deletion fragment, CSP7, delivered systemically by i.p. injection, by liquid nebulization, or by inhalation of micronized dry powder via airways in 3 different preclinical mouse models (25). A comprehensive biochemical, histological, and protein expression study further revealed that CSP or CSP7 inhibited the augmented glycolysis, indicated by relatively lower levels of total lactate content in the fibrotic lung tissues.

To support our observation that CSP or CSP7 regulates glucose metabolism by controlling p53 and miR-34a expression through feedback induction in fLfs, we performed a similar set of experiments with tamoxifen inducible *p53*^cKO^ or *miR34a*^cKO^ mice lacking p53 or miR-34a expression in fibroblasts. Because CSP or CSP7 failed to resolve existing lung fibrosis in *p53*^cKO^ (22) or *miR34a*^cKO^ (data not shown) mice, we tested these mice for changes in glycolysis. As we expected, CSP and CSP7 failed to suppress the expression of glycolytic enzymes in fibroblast-specific *p53*^cKO^ or *miR34a*^cKO^ mice, unlike WT or respective floxed mice. These observations support the contention that CSP or CSP7 regulates glucose metabolism by restoring baseline expression of p53 and miR-34a through feedback induction, and the expression of p53 and miR-34a is otherwise lost in fLfs. Elevated glycolysis could contribute to profibrogenic phenotypes often exhibited by fLfs as excessive proliferation and resistance to apoptosis. Increased secretion of intermediaries of the glycolytic pathway, such as lactate in alveolar epithelial cell dysplasia, are most frequently observed in type II alveolar epithelial cells surrounding fibrotic foci in IPF lungs (44).

HIF-1α is a master transcriptional regulator of cellular and developmental response to hypoxia. HIF-1α stimulates multiple glycolytic gene expression (45, 46). It has been shown that p53 activities are modulated by the inhibition of its translation by the HIF-1α–induced RNA binding proteins (47). It is known that HIF-1α and p53 display opposing effects on glycolysis and mitochondrial respiration. HIF-1α stimulates glycolysis by inducing the expression of the majority of glycolytic enzymes, as well as glucose and lactate transporters, whereas p53 represses glycolysis, which is consistent with our findings. HIF-1α has been shown to regulate the expression of glycolytic enzymes and key transcriptional factors that drive glycolysis under hypoxic conditions (45, 48). It is also well recognized that HIF-1α plays an important role in tissue fibrosis (49–51). The mutual influence of p53 and HIF-1α is highly contradictory. However, there is ample evidence to support the contention that HIF-1α–mediated degradation decreases p53 levels (52, 53). Elevated levels of HIF-1α downregulates homeodomain-interacting protein kinase-2 (HIPK2), a tumor suppressor that impair p53 activity (54). It is also reported that, under hypoxic condition, MDM2 is upregulated at the mRNA level (53, 55), which may lead to increased ubiquitination of p53. We have also reported that CSP/CSP7 directly binds to MDM2 in the absence of CAV1 and inhibits MDM2-mediated ubiquitination of p53 (22). miR-34a may regulate HIF-1α via inhibiting SIRT1 translation (28, 56). HIF-1α can also inhibit expression of miR-34a as reported in isogenic colorectal cancer cells (57). Inhibition of HIF-1α may contribute to reduced PFKFB3, which may have occurred because of inhibition of hypoxic conditions due to CSP7 intervention. Increased succinate and HIF-1α in fibrotic lung tissues suggests that CSP7-mediated suppression of HIF-1α may be linked to inhibition of the succinate activity. However, a recent report suggests that destabilization of HIF-1α has no effect on TGF-β1–induced PFKFB3 expression in lung fibroblasts (15). In addition, CSP7-mediated inhibition of α-SMA in fLfs (22, 25) may be associated with fLf dedifferentiation due to inhibition of HIF-1α, as the latter binds to the α-SMA promoter to induce its transcription. HIF-1α also induces transcription of glycolytic enzymes such as HK (58) and lactate dehydrogenase A (46), suggesting that CSP/CSP7-mediated inhibition of HK and lactate causally linked to suppression of HIF-1α. Furthermore, increased glycolysis observed in stromal fibroblasts that nourish surrounding tumor cells show reduced CAV1 expression like fLfs (59). Our results show that CSP/CSP7 restores basal expression of p53 (22) and miR-34a (data not shown), thereby regulating augmented glycolysis. Since p53 is known to transactivate miR-34a and the latter is involved in glucose metabolism (16), we speculated that p53 might directly interact with miR-34a to regulate the expression of glycolytic enzymes in lung fibroblasts. As expected, we found that glycolytic markers were regulated by p53 and miR-34a. In addition, both CSP and CSP7 inhibits AKT/PKB phosphorylation by dephosphorylation and activation of p53 transcriptional target and tumor suppressor protein PTEN (22), leading to reduced metabolic activation. Although proved in vitro, the effects of CSP/CSP7 on glycolysis in animal models still need further exploration. None of the mouse models fully recapitulate all the cardinal manifestations of human IPF. CSP/CSP7 may mitigate pulmonary fibrosis via acting on multiple cell types and attenuation of both inflammatory and fibrotic pathways in vivo (25, 60). Mitigation of lung fibrosis by CSP/CSP7 will alleviate tissue hypoxia, which could also inhibit HIF-1α and glycolysis in vivo.

In summary, we have demonstrated that p53 and its transcriptional target miR-34a regulate glycolysis in lung fibroblasts, and CSP7, when used as a therapeutic agent, ramps down the elevated key enzymes involved in glycolytic pathways in multiple preclinical mouse models of established pulmonary fibrosis and in isolated hfLfs and mfLfs via restoration of p53 and miR-34a expression. These observations suggest that CSP7 may be a candidate for further development as a therapeutic agent to restrain runaway glycolysis of fLfs required for the initiation and sustainability of the profibrogenic phenotype of these cells that contributes to progressive lung fibrosis.

## Methods

### Cell isolation and culture.

Human nLfs and fLfs (IPF) were purchased from Cory Hogaboam (University of Michigan, Ann Arbor, Michigan, USA), provided by Ganesh Raghu (University of Washington, Seattle, Washington, USA), or isolated locally from control and IPF lung tissues as described elsewhere (22, 25, 61). All hfLfs used in the study were well characterized, derived from patients with usual interstitial pneumonia, as described (22, 61). To avoid heterogeneity associated with fLfs, we selected fLfs expressing elevated levels of profibrogenic marker proteins such as collagen-1α, α-SMA, fibronectin, and tenascin-C with minimal expression of CAV1, p53, and PAI-1 as we described earlier (22, 61). Human nLfs were derived from control donors without apparent interstitial or other lung diseases. Mouse nLfs were isolated from the lungs of mice without any lung injury and fLfs from the fibrotic lungs of mice 21 days after exposure to BLM. Primary lung fibroblasts were isolated as described (25, 61). Briefly, human or mouse lung tissues were minced and digested with 0.1% collagenase and 0.005% trypsin in HBSS for 1 hour at 37°C. Fibroblasts were selected by adherence of the suspension on cell culture dishes. The adherent lung fibroblasts were tested for profibrogenic phenotypes by analyzing collagen 1α, α-SMA, fibronectin, and tenascin-C, as well as CAV1 and p53 expression; cultured in DMEM containing 4.5 g/L glucose, 10% FBS, and 1% penicillin and streptomycin at 37°C; and used within 3–5 passages of initial isolation.

### Lactate and succinate production measurement.

Lactate and succinate levels were measured using lactate (catalog K627) colorimetric assay kit II and succinate colorimetric assay kit (catalog K649), respectively (BioVision) according to the manufacturer’s instructions.

### Western blotting of cell lysates and lung homogenates.

Western blotting was performed as previously described (22, 43, 62). Rabbit anti-HK2 (catalog C64G5), -PKM (catalog D78A4), -PFKP (catalog D4B2), –HIF-1α (catalog D2U3T), and -PFKFB3 (catalog D7H4Q) antibodies were purchased from Cell Signaling Technology. Mouse anti-p53 (catalog SC55476) antibody was purchased from Santa Cruz Biotechnology Inc.

### Isolation and analysis of RNA by qPCR.

Total RNA, isolated from cells or whole lung homogenates treated with or without CSP, CSP7, or CP, as described above, was reverse transcribed and subjected to qPCR for *HK2*, *PKM*, *PFKP*, *PFKFB3*, and *HIF1A* transcripts (22). Primer sequences used in this study have been listed in Table 1.

### H&E staining and IHC analyses of lung sections.

These analyses were performed as we previously reported (22, 43).

### Overexpression and inhibition of p53 or miR-34a in fibroblasts in vitro.

hfLfs or mfLfs were transduced with Ad-p53 or Lv–Pre–miR-34a. Naive fLfs and fLfs transduced with Ad-Ev or Lv-Ev were used as controls. In a separate experiment, nLfs in culture dishes were treated with Lv-p53 shRNA or Lv–miR-34a–As to suppress their baseline expression. Naive nLfs and nLfs exposed to nonspecific Ctrl shRNA were used as controls.

### Mice.

Male and female C57BL/6 mice (8–10 weeks old) were purchased from The Jackson Laboratory. For generation of inducible *p53*^cKO^ or *miR34a*^cKO^ mice lacking expression in fibroblasts, *p53^fl/fl^* or *miR34a^fl/fl^* mice were cross-bred with *Col*^Cre^ mice. First-generation heterozygous *fl* and *Col*^Cre^ mice were back crossed with homozygous *fl/fl* mice. The pups carrying only mutant *p53* or *miR34a* and *Col*^Cre^ alleles were consecutively given i.p. injection of tamoxifen (75 mg/kg body weight) once every 24 hours for 5 consecutive days to inhibit p53 or miR-34a expression in fibroblasts as described (22). The *p53^fl/fl^* or *miR34a^fl/fl^* and *p53*^cKO^ or *miR34a*^cKO^ mice were exposed to BLM to induce pulmonary fibrosis.

### Single-dose BLM-induced pulmonary fibrosis model.

WT, *p53^fl/fl^*, *p53*^cKO^, *miR34a^fl/fl^*, and *miR34a*^cKO^ mice were exposed once to freshly prepared BLM (8 U/kg) (catalog RB 003, TSZCHEM) in 50 μL saline or only saline through intranasal instillation. Two weeks after BLM exposure, mice were treated with peptides (1.5 mg/kg/d) for 7 days as we described earlier (22, 25).

### TGF-β1–induced pulmonary fibrosis model.

WT mice were exposed to saline or 1 × 10^9^ plaque forming units (pfu) of Ad-Ev or Ad–TGF-β1. Two weeks later, mice were treated daily with peptide for 14 days as we described (25, 63).

### Multiple-dose BLM-induced pulmonary fibrosis model.

WT mice were exposed to BLM (2 U/kg) in 50 μL saline or only saline through intranasal instillation once every 2 weeks, 8 times. Two weeks after the eighth dose, BLM mice were treated with CSP/CSP7 once daily for 2 weeks, as we described (25).

### Statistics.

The differences between 2 groups were analyzed by 2-tailed Student’s *t* test and between multiple groups using 1-way ANOVA followed by Tukey’s post hoc test. *P* < 0.05 was considered statistically significant.

### Study approval.

All animal experiments reported here were approved by the UTHCT IACUC. The human protocol was approved by the UTHCT IRB.

## Author contributions

VG, LF, RSS, and MRN performed the experiments. SS conceived idea and designed the experiments. VG and SS wrote the manuscript.

## Figures and Tables

**Table 1:**
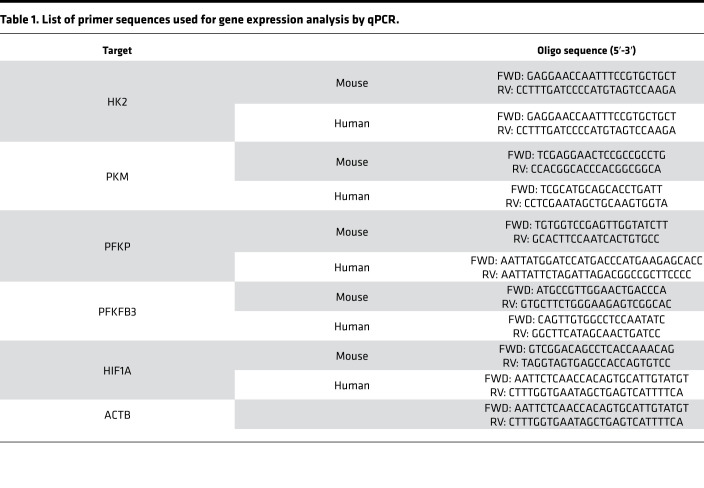
List of primer sequences used for gene expression analysis by qPCR.

## Version 1 08/25/2020

In-Press Preview

## Version 2 10/02/2020

Electronic publication
